# Effects of Rotational Speed on Joint Characteristics of Green Joining Technique of Dissimilar Polymeric Rods Fabricated by Additive Manufacturing Technology

**DOI:** 10.3390/polym14224822

**Published:** 2022-11-09

**Authors:** Chil-Chyuan Kuo, Hong-Wei Chen, Jing-Yan Xu, Chong-Hao Lee, Song-Hua Hunag

**Affiliations:** 1 Department of Mechanical Engineering, Ming Chi University of Technology, No. 84, Gungjuan Road, New Taipei City 243, Taiwan; 2 Research Center for Intelligent Medical Devices, Ming Chi University of Technology, No. 84, Gungjuan Road, New Taipei City 243, Taiwan; 3 Li-Yin Technology Co., Ltd., No. 37, Lane 151, Section 1, Zhongxing Road, Wugu District, New Taipei City 241, Taiwan

**Keywords:** friction welding, material extrusion process, green products, mechanical properties

## Abstract

Friction welding (FW) FW of dissimilar polymer rods is capable of manufacturing green products swiftly and economically. In this study, a green manufacturing technique of joining dissimilar polymer rods was proposed, and the effects of rotational speed on the joint characteristics of friction-welded dissimilar polymer rods fabricated by the fused deposition modeling process were investigated experimentally. The shore surface hardness test, impact test, three-point bending test, and differential scanning calorimetry analysis were carried out on the weld joints. The impact energy for FW of polylactic acid (PLA) and PLA, PLA and acrylonitrile butadiene styrene (ABS), PLA and PLA filled with glass fiber (GF), PLA and PLA filled with carbon fiber (CF), PLA and polycarbonate (PC), and PLA and polyamide (PA) rods can be increased by approximately 1.5, 1.5, 1.3, 1.3, 2.1, and 1.5 times by increasing the rotational speed from 330 rpm to 1350 rpm. The bending strength for FW of PLA and PLA, PLA and ABS, PLA and PLA filled with GF, PLA and PLA filled with CF, PLA and PC, and PLA and PA rods can be increased by approximately 1.3, 1.7, 1.3, 1.2, 1.2, and 1.2 times by increasing the rotational speed from 330 rpm to 1350 rpm. However, the surface hardness of the weld bead is not proportional to the rotational speed. The average surface hardness of the weld bead was increased by approximately 5% compared to the surface hardness of the welding base materials.

## 1. Introduction

Friction welding (FW) [1] is widely used as a mass-production method in various industries because it is a non-fusion welding process. In practice, FW has been used in railways, aerospace, automobile, automotive, chemical, and marine industries because it generates heat through mechanical friction between parts in relative motion. FW has some distinct advantages, such as the short welding time, high efficiency, and absence of shielding gas [2,3]. In general, FW of dissimilar materials [4,5] has many applications in academic research and industrial applications. Wang et al. [6] studied the rotary FW on dissimilar brass using the innovative pre-heating method. The microstructure examination revealed that a narrow intermetallic compound layer was formed, showing metallic bonding on the interface. Hynes et al. [7] proposed a predictive thermal distribution model during FW of ceramics with metal. The proposed simulation model provided the potential prediction of the formation of residual stress in the mild steel-alumina side of the interface. Pereira et al. [8] investigated the influence of different welding parameters on the morphology and mechanical strength of friction stir welds in polymers. The utilization of tools with a stationary shoulder and external heating provided great interest to improve the surface finish and increase the mechanical strength of welds. Winiczenko et al. [9] studied the effects of FW process parameters on the microstructural properties of dissimilar joints. It was found that the maximum tensile strength of the friction-welded low-carbon steel-ductile iron joints is approximately 87% of that of the base metal. Hangai et al. [10] investigated the effects of the porosity of aluminum foam on welding. The aluminum foam can be welded to a polycarbonate plate by FW with the pore structures retained. Ezzat et al. [11] investigated the effects of surface preparation on the strength of a vibration-welded butt joint made from a polybutylene terephthalate composite. It was found that fiber orientation is a major factor affecting the strength of the joints. Elsheikh et al. [12] discussed the application of these composites in energy harvesting. Results showed that the bistability response of the composite laminates was greatly affected by the thermal expansion coefficient, temperature variation, moisture content, coefficient of moisture, and laminate thickness. Elsheikh et al. [13] proposed a new hybrid artificial intelligence approach to model the ultrasonic welding of a polymeric material blend. In addition, four models were also investigated using five statistical tools. Kamal et al. [14] reviewed the fabrication techniques of a polymeric nanocomposite. In addition, the working principles of each technique are discussed.

Three-dimensional printing (3DP) [15,16] is particularly useful for manufacturing functional components or prototypes. Among the 3DP technique, the fused deposition modeling (FDM) process [17,18] is frequently used to fabricate functional components or prototypes with complex geometries because of the wide range of materials that can be used with this technique, such as polyamide (PA) [19], polylactic acid (PLA) [20], polycarbonate (PC) [21], or acrylonitrile butadiene styrene (ABS) [22,23,24]. The advantages of adhesive bonding [25] involve fast and cheap joining providing design flexibility and thin and invisible joints and the uniform distribution of mechanical stress over the joint. Disadvantages of adhesive bonding include the low creep strength, long mixing and curing time requirements, and the necessity to fix the joined parts during curing. Fusion bonding [26] has the advantage of being able to bond materials together without considering the coefficient of thermal expansion of the workpiece. According to practice experience, FW of dissimilar thermoplastic materials is one of the best solutions in the industry due to the high welding efficiency and welding effectiveness [27]. However, FW of dissimilar thermoplastic is a tricky task because the material flow during FW is one of the factors influencing the joint strength required for manufacturing qualified welded parts, especially in the different rotational speeds used in FW of dissimilar thermoplastic materials. Thus, the investigation of the effects of rotational speed on the joint strength of welded parts fabricated by 3DP is an important research topic. In this study, a green manufacturing technique used to join dissimilar polymer rods was proposed, and we investigated the effects of the rotational speed on the joint characterization of welded parts. Six different kinds of thermoplastic materials are employed to print cylindrical rods. An infrared thermal imager is used to investigate the peak temperature of the weld joint during FW under different rotational speeds. After FW, optical microscopy (OM) and field-emission scanning electron microscopy (FE-SEM) were used to study the microstructure of the weld joints. The multiple-step transformation behavior of weld joints was evaluated using differential scanning calorimetry (DSC) analysis. To determine the joint characteristics of welded parts after FW, surface hardness tests, impact tests [28], and three-point bending tests [29,30] were carried out. Finally, an empirical technical database of the FW strength of polymer rods under different rotational speeds is proposed in this study.

## 2. Experimental Details

**Figure 1:**
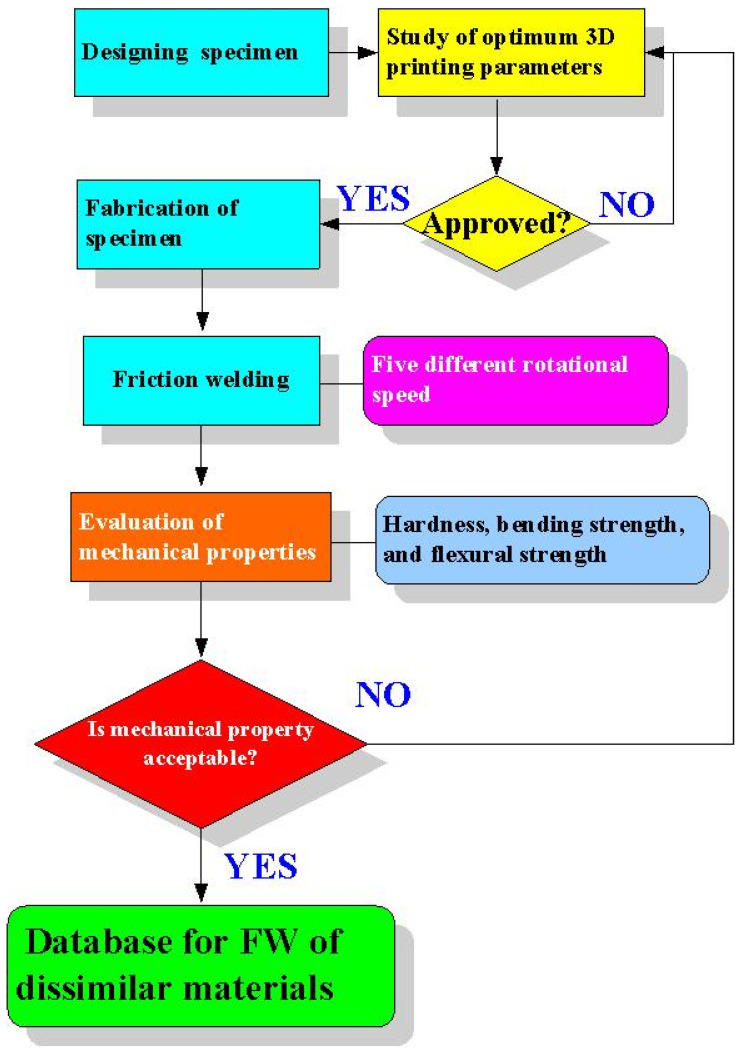
Flow diagram of the experimental methodology.

**Figure 2:**
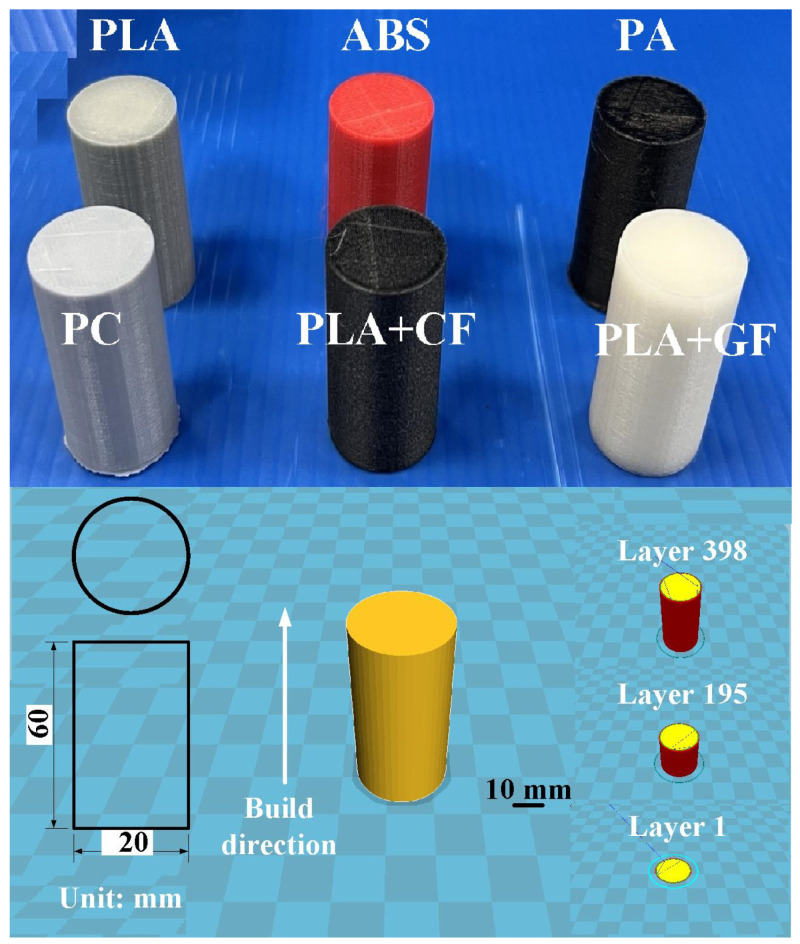
CAD model and dimensions of FW specimens.

Figure 1 shows the flow diagram of the experimental methodology. The research process involves designing the specimen, investigating the optimum 3D printing parameters, fabricating specimens, FW, evaluating the mechanical properties of the welded parts, and establishing a database for FW of dissimilar materials. Firstly, the specimen was designed as a cylindrical rod with a diameter of 20 mm and a length of 40 mm. Figure 2 shows the CAD model and dimensions of FW specimens. The software named ultimaker Cura was used to generate a printing program for FW specimens. The weld specimen was placed vertically for printing. In this study, six different kinds of thermoplastic materials, i.e., PLA (Thunder 3D Inc., Taipei, Taiwan), PLA filled with 10 wt.% glass fiber (GF) (Thunder 3D Inc., Taipei, Taiwan), PLA filled with 10 wt.% carbon fiber (CF) (Thunder 3D Inc., Taipei, Taiwan), ABS (Thunder 3D Inc., Taipei, Taiwan), PC, and PA were used to print FW specimens using an FDM 3D printer (Mini-200, Teklink smart solution Inc., New Taipei City, Taiwan). The printing parameters for PA and PC filaments include a printing temperature of 240 °C, a printing speed of 50 mm/s, a layer thickness of 0.1 mm, and a printing bed temperature of 100 °C. The printing parameters for the ABS filament include a printing temperature of 225 °C, a printing speed of 45 mm/s, a layer thickness of 0.1 mm, and a printing bed temperature of 100 °C. The printing parameters for the PLA filament include a printing temperature of 210 °C, a printing bed temperature of 65 °C, a printing speed of 75 mm/s, and a layer thickness of 0.1 mm. The infill density was fixed at 100%. The printing parameters for PLA filled with 10 wt.% GF filaments include a printing temperature of 210 °C, a printing bed temperature of 75 °C, a printing speed of 75 mm/s, and a layer thickness of 0.1 mm. The printing parameters for PLA filled with 10 wt.% CF filaments include a printing temperature of 210 °C, a printing bed temperature of 75 °C, a printing speed of 75 mm/s, and a layer thickness of 0.1 mm.

**Figure 3:**
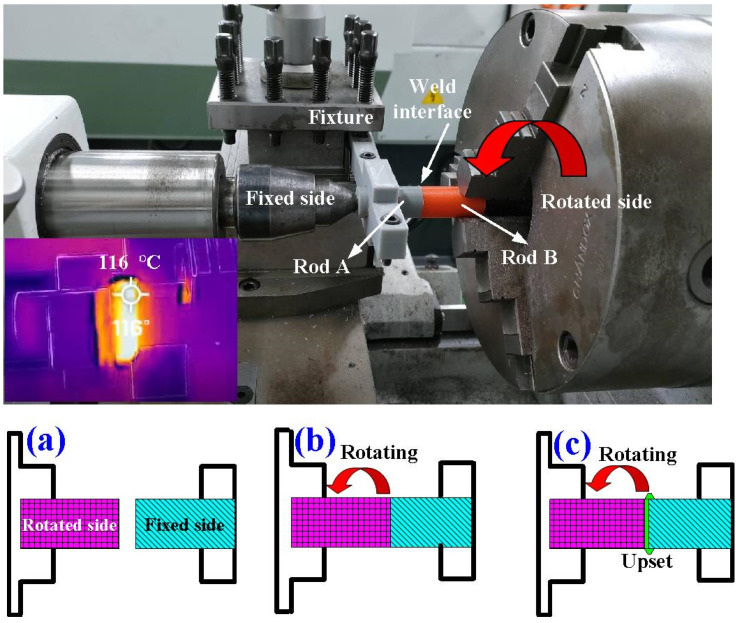
Situation of FW and schematic illustration of FRW process. (**a**) Installing two welding specimens in the conventional turning machine, (**b**) rotating one welding specimen, and (**c**) applying pressure to force FRW specimens to contact.

Polymer FW is a solid-state joining process, which provides axial movement to obtain the required weld strength. During FW, axial pressure is used for the consolidation of the weld. One polymer rod was rotated at a constant speed while the other was kept stationary. Two polymer rods were brought together under pressure for a certain period of time. Table 1 shows the experimental conditions of FW. In this study, the cycle time of FW was set to 60 s according to previous work [31]. The cycle time involves a friction time of 30 s, a forge time of 20 s, and a cooling time of 10 s. The burn-off length was set to 2 mm since the FW was carried out 20 times with a weld length of 0.1 mm each time. To investigate the effects of the rotational speed on the joint strength of friction-welded dissimilar polymer rods fabricated by three-dimensional printing, five different rotational speeds, i.e., 330 rpm, 490 rpm, 650 rpm, 950 rpm, and 1350 rpm were employed in this study. Figure 3 shows the situation of FW and a schematic illustration of the FRW process. A conventional lathe was used to perform FW. One cylindrical rod is kept stationary while the other is rotated at a constant speed. In this study, a fixture is designed and implemented to clamp one cylindrical rod to prevent one rod from simultaneously rotating with the rotating cylindrical rod. In general, the heat generated by friction in the weld joint during FW emits infrared energy [32]. The peak temperature of the weld bead during FW was monitored and recorded using an infrared camera (BI-TM-F01P, Panrico trading Inc., New Taipei City, Taiwan).

After FW, the mechanical properties of welded parts were determined. The weld joint was investigated using an OM (Quick Vision 404, Mitutoyo Inc, Tokyo, Japan). DSC (STA 409 PC Luxx Simultaneous thermal analyzer, Netzsch-Gerätebau GmbH Inc., Selb, Germany) was used to estimate the melting and mesomorphic transitions along with the entropy and enthalpy of the weld joints. The heating and cooling rate of the DSC test samples was approximately 15 °C/min under the gas flow of nitrogen of approximately 25 mL/min. The flexural strength of welded parts was investigated using a three-point bending test machine (RH-30, Shimadzu Inc., Kyoto, Japan) with a movement speed of approximately 1 mm/s. The impact energy of welded parts was investigated using a Charpy impact testing machine (780, Instron Inc., Norwood, MA, USA).

## 3. Results and Discussion

**Figure 4:**
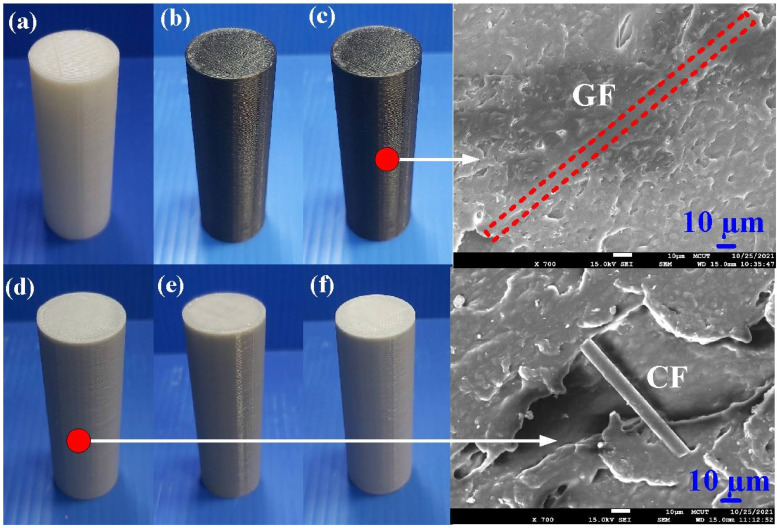
Polymer rods built with (**a**) PLA, (**b**) ABS, (**c**) 10% GF-reinforced PLA, (**d**) 10% CF-reinforced PLA, (**e**) PA, and (**f**) PC feedstock using FDM process.

**Figure 5:**
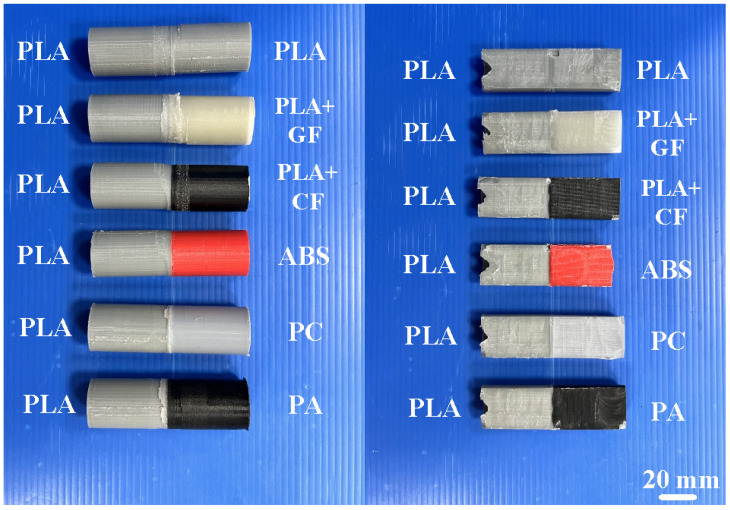
Friction-welded dissimilar polymer rods of whole and half proportions.

In this study, PLA is used as the main material for FW due to its fabrication from renewable raw materials. In addition, PLA melts easily due to its lower melting point compared to many fossil-based plastics. Therefore, it is easy to weld with PLA in terms of having less energy to transform. Figure 4 shows the polymer rods built with PLA, ABS, 10% GF-reinforced PLA, 10% CF-reinforced PLA, PA, and PC feedstock using the FDM process. Insets show the FE-SEM micrographs of PLA filled with 10 wt.% GF and PLA filled with 10 wt.% CF. Figure 5 shows the friction-welded dissimilar polymer rods in whole and half proportions.

**Figure 6:**
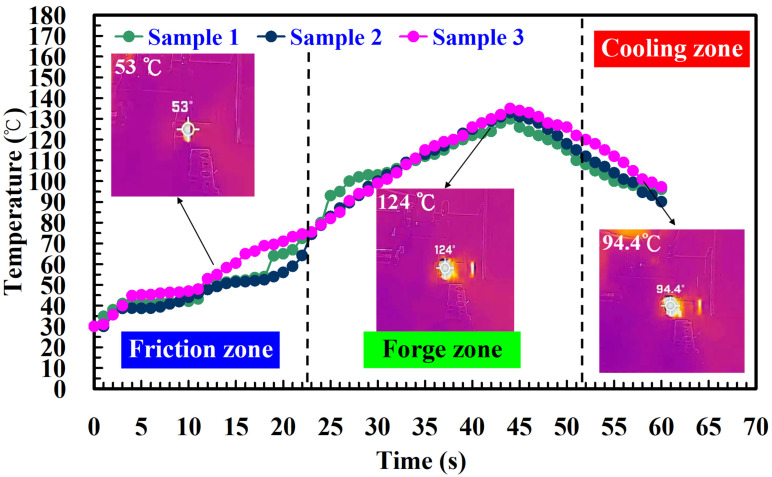
The relationship between weld-joint temperature and FW time for PLA and PLA rods at a rotational speed of 1350 rpm.

**Figure 7:**
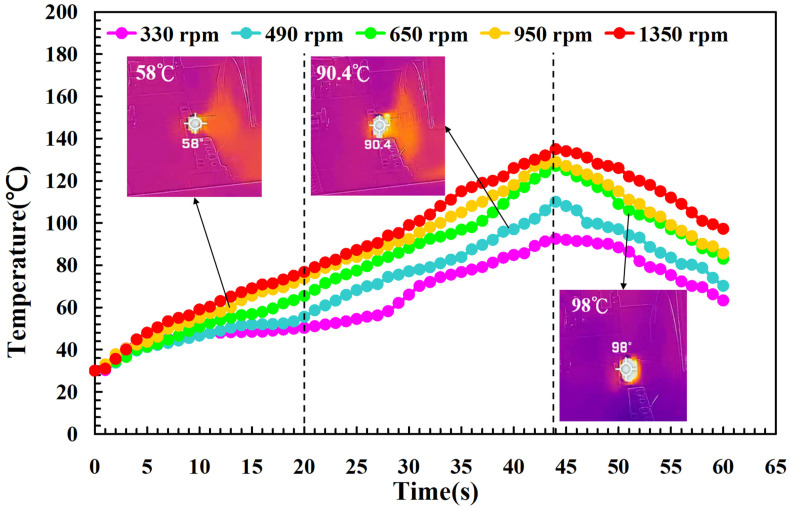
The relationship between weld-joint temperature and FW time for the PLA and PLA rods at five different rotational speeds.

**Figure 8:**
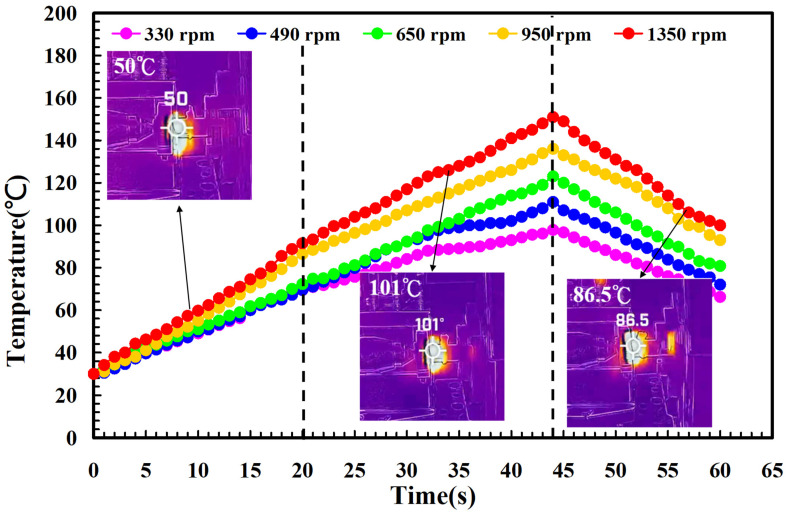
The relationship between weld-joint temperature and FW time for the PLA and PLA filled with GF rods at five different rotational speeds.

**Figure 9:**
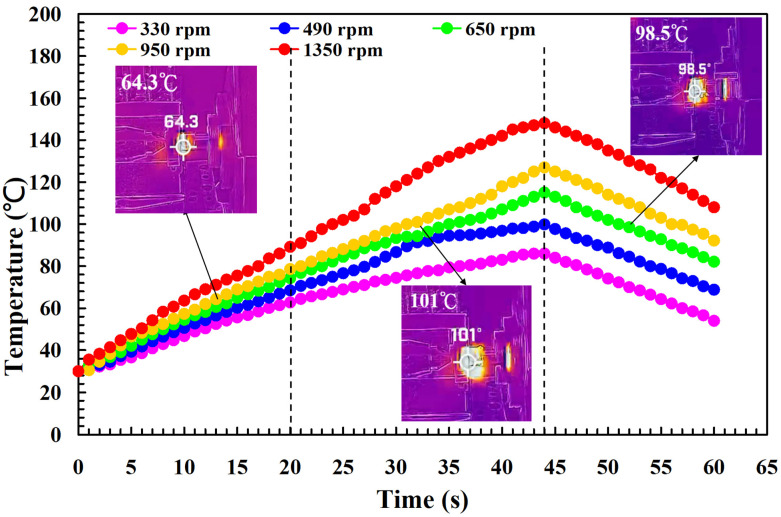
The relationship between weld-joint temperature and FW time for the PLA and PLA filled with CF rods at five different rotational speeds.

**Figure 10:**
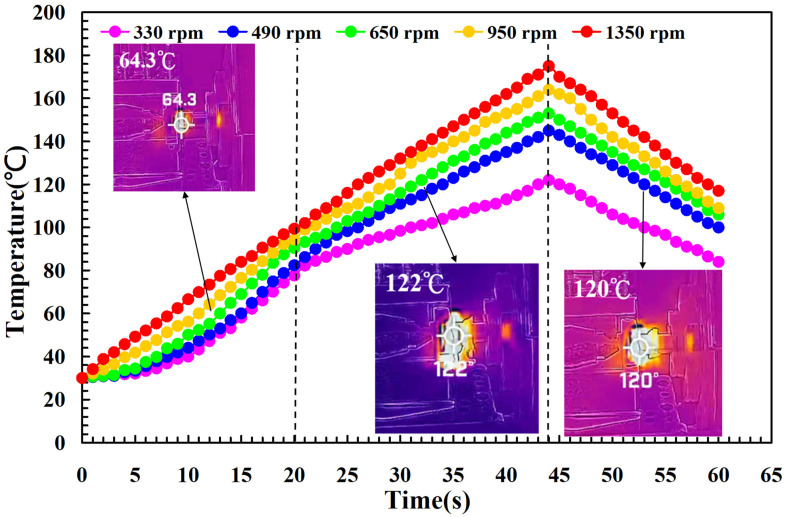
The relationship between weld-joint temperature and FW time for the PLA and ABS rods at five different rotational speeds.

**Figure 11:**
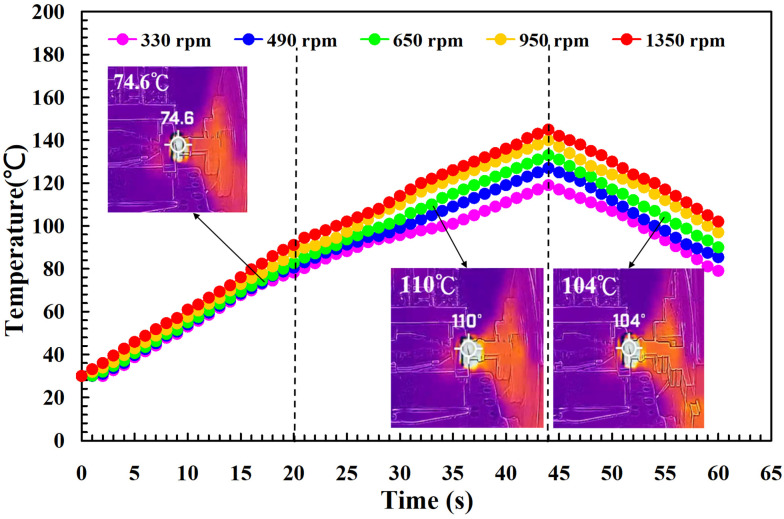
The relationship between weld-joint temperature and FW time for the PLA and PC rods at five different rotational speeds.

**Figure 12:**
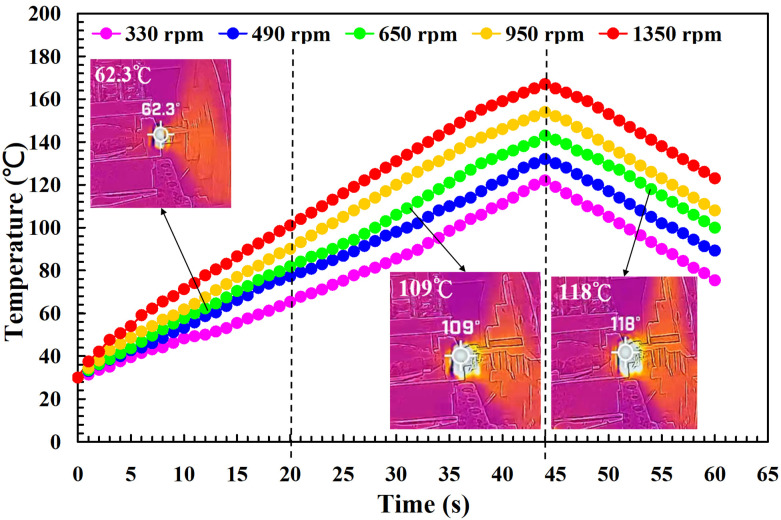
The relationship between weld-joint temperature and FW time for the PLA and PA rods at five different rotational speeds.

**Figure 13:**
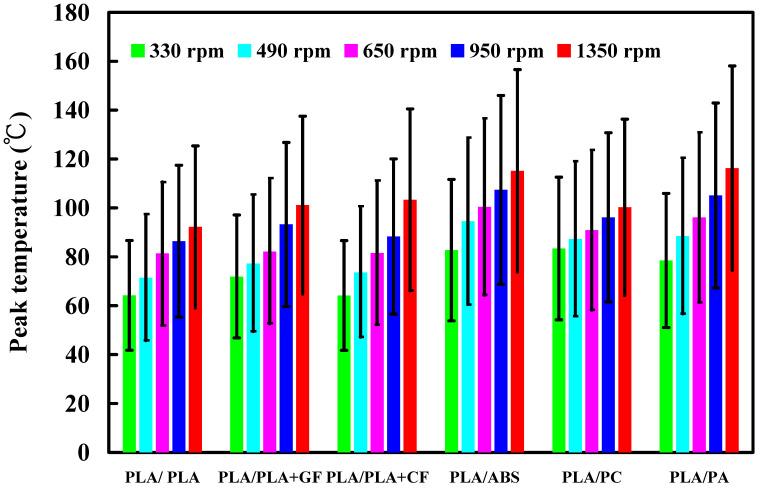
The highest and lowest peak temperatures for FW of six dissimilar materials at five different rotational speeds.

To investigate the influence of human factors on the FW, the experiments were repeated three times for PLA and PLA rods. Figure 6 shows the relationship between the weld-joint temperature and FW time for PLA and PLA rods at a rotational speed of 1350 rpm. Two phenomena were found. One is that the relationship between the weld-joint temperature and FW time for PLA and PLA rods in FW at a rotational speed of 1350 rpm is repeatable. The other is that the peak temperature of the weld joint is approximately 135 °C. Figure 7 shows the relationship between the weld-joint temperature and FW time for PLA and PLA rods at five different rotational speeds. The results showed that the peak temperatures of the weld joint at rotational speeds of 330 rpm, 490 rpm, 650 rpm, 950 rpm, and 1350 rpm are approximately 93 °C, 110 °C, 127 °C, 129 °C, and 135 °C, respectively. It was found that the peak temperatures of the weld joint gradually increase with an increased rotational speed. This result indicates that a higher weld-joint temperature results from a higher rotational speed. Figure 8 shows the relationship between the weld-joint temperature and FW time for the PLA and PLA filled with GF rods at five different rotational speeds. Figure 9 shows the relationship between the weld-joint temperature and FW time for the PLA and PLA filled with CF rods at five different rotational speeds. Figure 10 shows the relationship between the weld-joint temperature and FW time for the PLA and ABS rods at five different rotational speeds. Figure 11 shows the relationship between the weld-joint temperature and FW time for PLA and PC rods at five different rotational speeds. Figure 12 shows the relationship between the weld-joint temperature and FW time for the PLA and PA rods at five different rotational speeds. Figure 13 shows the highest and lowest peak temperatures for FW of six dissimilar materials at five different rotational speeds. The results showed that the lowest peak temperatures of FW for the PLA and PLA, PLA and ABS, PLA and PLA filled with GF, PLA and PLA filled with CF, PLA and PC, and PLA and PA rods are round 93 °C, 122 °C, 98 °C, 86 °C, 119 °C, and 122 °C, respectively. In addition, the highest peak temperatures of FW for the PLA and PLA, PLA and ABS, PLA and PLA filled with GF, PLA and PLA filled with CF, PLA and PC, and PLA and PA rods are approximately 134 °C, 175 °C, 151 °C, 148 °C, 145 °C, and 167 °C, respectively. The results showed that the bead temperature for FW of six dissimilar materials increased with an increased rotational speed.

**Figure 14:**
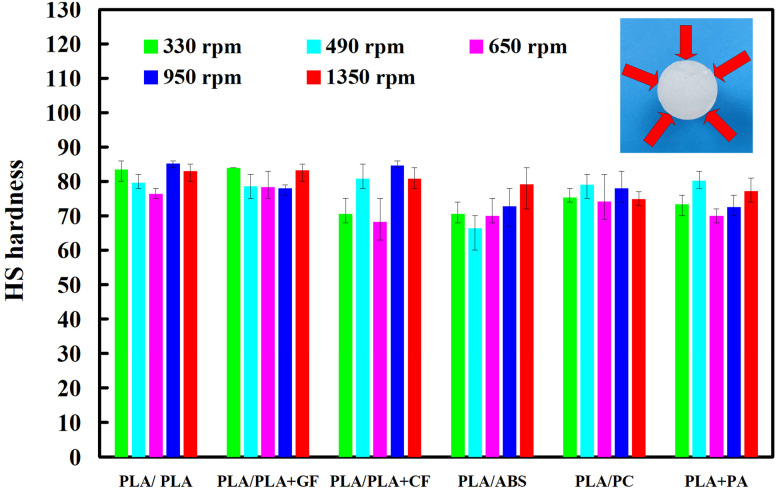
Surface hardness of weld joint of dissimilar polymer rods welded at five different rotational speeds.

The average shore hardness of the welding base materials of PLA, ABS, PLA filled with GF, PLA filled with CF, PC, and PA are approximately HS 78.2, HS 74.4, HS 78.4, HS 73, HS 77 and HS 76.8, respectively. Figure 14 shows the surface hardness of the weld joint of dissimilar polymer rods welded at five different rotational speeds. It was found that the average surface hardness of the weld bead increased by approximately 5% compared with the surface hardness of the welding base materials. Three phenomena were found: (a) The average surface hardness of the weld bead is not proportional to the rotational speeds, (b) the surface hardness of PLA filled with GF is the highest and the surface hardness of PLA and PLA filled with CF is the lowest, and (c) the surface hardness of PLA is higher than ABS.

**Figure 15:**
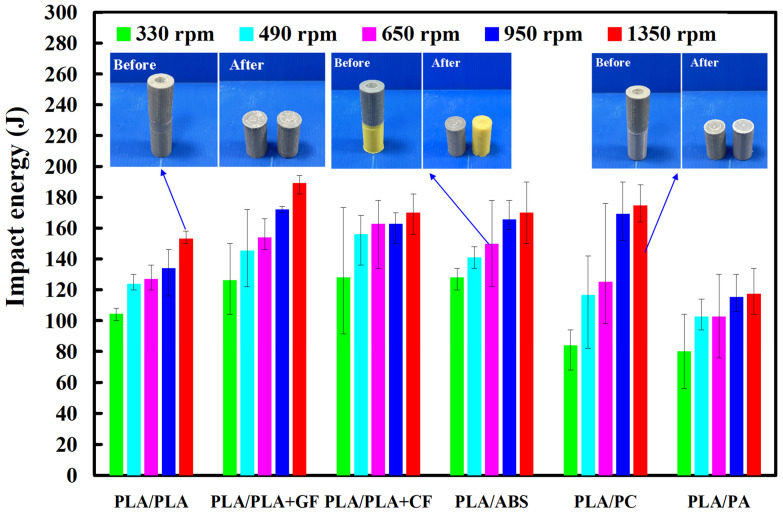
Impact energy of dissimilar polymer rods welded at five different rotational speeds.

**Figure 16:**
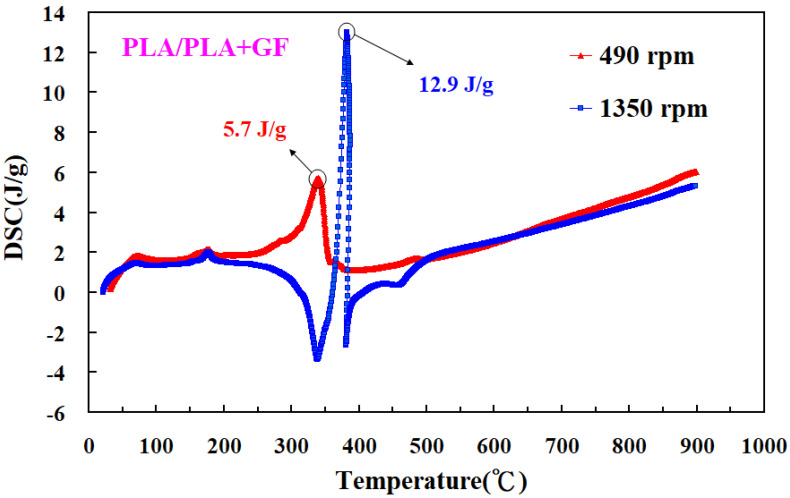
DSC curves for samples welded at 490 rpm and 1350 rpm.

**Figure 17:**
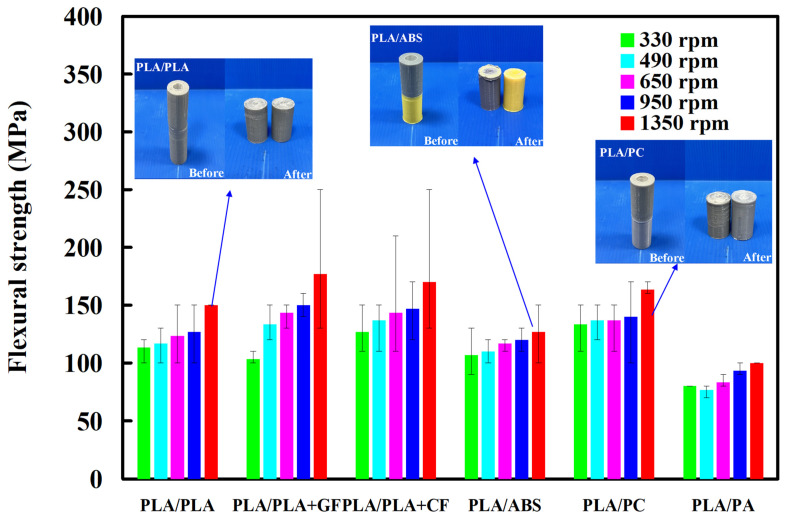
Flexure strength of dissimilar polymer rods welded at five different rotational speeds.

Figure 15 shows the impact energy of dissimilar polymer rods welded by five different rotational speeds. Three phenomena were found: (a) The impact energy of the six dissimilar 3D-printed parts after FW increased with an increase in the rotational speed. It should be noted that the impact energy for FW of PLA and PLA, PLA and ABS, PLA and PLA filled with GF, PLA and PLA filled with CF, PLA and PC, and PLA and PA rods can increase by approximately 1.5, 1.5, 1.3, 1.3 2.1, and 1.5 times by increasing the rotational speed from 330 rpm to 1350 rpm. The main reason is that a higher rotational speed resulted in a higher temperature in the weld joints, resulting in better molecular orientation in the weld joints after FW. This result is confirmed by the DSC test [33] because the normalized heat capacities for the samples welded at 490 rpm and 1350 rpm are 5.7 J/g and 12.9 J/g, respectively. Furthermore, (b) the impact energy for dissimilar FW of PLA and PLA filled with CF is the highest, and (c) the impact energy for dissimilar FW of PLA and PLA filled with PA is the lowest. Figure 16 shows the DSC curves for samples welded at 490 rpm and 1350 rpm. Figure 17 shows the flexure strength of dissimilar polymer rods welded by five different rotational speeds. It should be noted that the flexure strength for FW of PLA and PLA, PLA and ABS, PLA and PLA filled with GF, PLA and PLA filled with CF, PLA and PC, and PLA and PA rods can be increased by approximately 1.3, 1.7, 1.3, 1.2, 1.2, and 1.2 times by increasing the rotational speed from 330 rpm to 1350 rpm.

**Figure 18:**
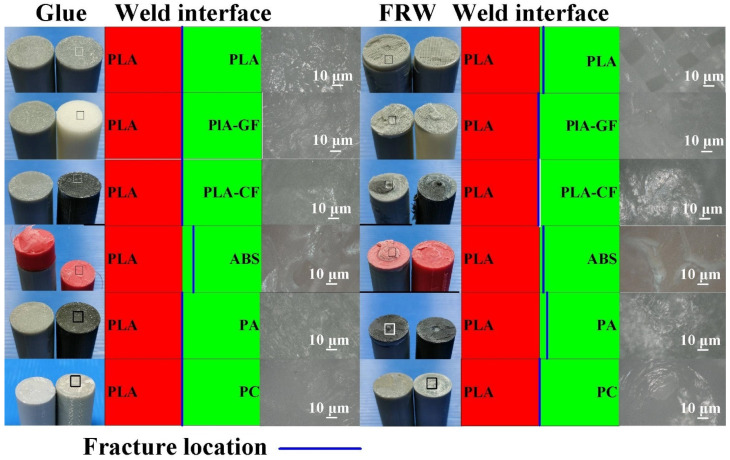
Optical microscopic images of fractured surfaces after bending test.

**Figure 19:**
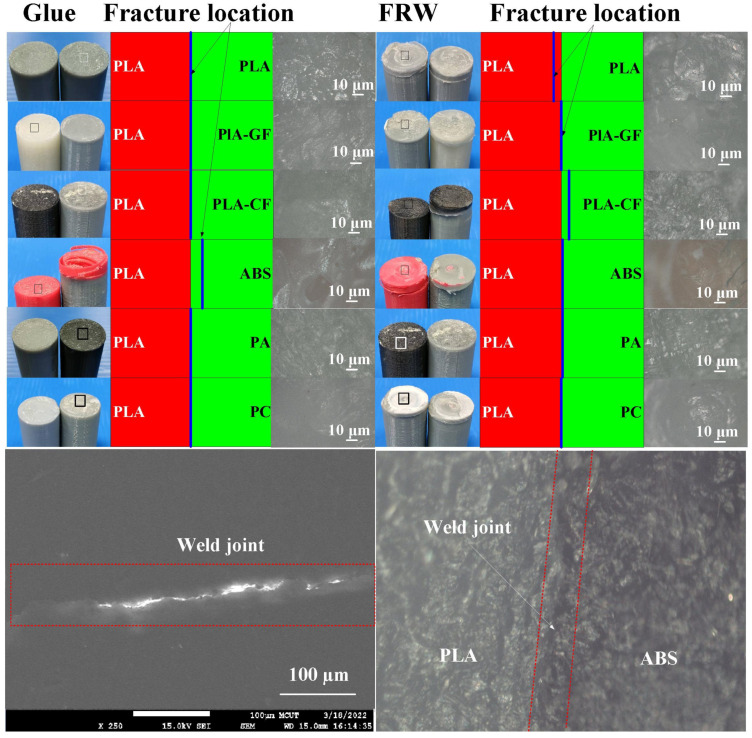
Optical microscopic images of fractured surfaces after impact test.

Figure 18 shows the optical microscopic images of fractured surfaces after the bending test. Two phenomena were found: (a) Most of the fracture locations are located in the weld joint after the bending test and (b) the fracture location is not located in the weld joint for FW of PLA and ABS rods and PLA and PA prods after the bending test. Figure 19 shows the optical microscopic images of fractured surfaces after the impact test. Two phenomena were found: (a) Most of the fracture locations are located in the weld joint after the impact test and (b) the fracture location is not located in the weld joint for FW of PLA and PLA rods and PLA and PLA filled with CF rods after the impact test. One possible reason is that the two materials have different rheological properties [34], such as the melt flow index [35] or glass transition temperature [36]. However, this study did not investigate this issue in depth. Therefore, these issues are important research directions for the future.

According to the experimental analysis described above, the specific findings provide the greatest application potential in the polymer welding industry. FW of dissimilar polymer rods is a green manufacturing technique joining dissimilar polymer rods and meets sustainable development goal 12 [37,38]. In general, this technique can be used for joining axle shafts, fluid mechanical components [39], aircraft components, aerospace components, automotive components, or transmission shafts [40]. In this study, the tensile strength of the welded part was not assessed. In addition, the molecular orientation of the welded zone was not evaluated. PLA has some distinct advantages, including its inexpensive costs, low melting point, easy printing, and lack of fumes. However, the mechanical properties of PLA [41] are lower than those of ABS [42] or PC [43]. Thus, the main material for FW could also be ABS or PC. In addition, the optimization of process parameters of FW of dissimilar polymeric rods using the design of experiments approach [44,45] is also an interesting research topic. These are interesting research topics and are currently being investigated, for which the results will be presented in later works.

## 4. Conclusions

Rotary FW is a solid-state welding process that generates heat through mechanical friction between thermoplastics or metals. Friction welding is used with metals and thermoplastics in a wide variety of aviation and automotive applications. The main conclusions from the experimental work in this study are as follows:

1. The bead temperature of FW was increased by increasing the rotational speed.
2. The impact energy of the welded parts of dissimilar materials was increased by increasing the rotational speed. The impact energy for FW of PLA and PLA, PLA and ABS, PLA and PLA filled with GF, PLA and PLA filled with CF, PLA and PC, and PLA and PA rods can be increased by approximately 1.5, 1.5, 1.3, 1.3, 2.1, and 1.5 times by increasing the rotational speed from 330 rpm to 1350 rpm.
3. The bending strength of the welded parts of dissimilar materials was increased by increasing the rotational speed. The bending strength for FW of PLA and PLA, PLA and ABS, PLA and PLA filled with GF, PLA and PLA filled with CF, PLA and PC, and PLA and PA rods can be increased by approximately 1.3, 1.7, 1.3, 1.2, 1.2, and 1.2 times by increasing the rotational speed from 330 rpm to 1350 rpm.
4. The surface hardness of the weld bead is not proportional to the rotational speed. However, the average surface hardness of the weld bead was increased by approximately 5% compared with the surface hardness of the welding base materials.

## Figures and Tables

**Table 1:** Experimental conditions of FW.

Process Parameters	Value
Friction time (s)	30
Forge time of (s)	20
Cooling time (s)	10
Rotational speed (rpm)	330, 490, 650, 950, and 1350

## Data Availability

The data presented in this study are available on request from the corresponding author.
